# Clinical performance of an alkasite-based bioactive restorative in class II cavities: a randomized clinical trial

**DOI:** 10.1590/1678-7757-2023-0025

**Published:** 2023-06-23

**Authors:** Fatma Dilsad OZ, Ece MERAL, Sevil GURGAN

**Affiliations:** 1 Hacettepe University School of Dentistry Department of Restorative Dentistry Ankara Turkey Hacettepe University, School of Dentistry, Department of Restorative Dentistry, Ankara, Turkey.

**Keywords:** Composite resins, Permanent dental restoration, Randomized controlled trial

## Abstract

**Objective:**

This clinical study aimed to evaluate the clinical performance of an alkasite-based bioactive material by comparing it with a resin composite (RC) in the restoration of Class II cavities over a year.

**Methodology:**

A hundred Class II cavities were restored at 31 participants. Groups were as follows: Cention N (CN) (Ivoclar Vivadent, Schaan, Liechtenstein) and G-ænial Posterior (GP) (GC, Tokyo, Japan) in combination with G-Premio Bond (etch&rinse). Restorative systems were applied following manufacturers’ instructions. They were finished and polished immediately after placement and scored based on retention, marginal discoloration, marginal adaptation, sensitivity, surface texture, and color match using modified USPHS criteria after 1 week (baseline), 6 months, and 12 months. Statistical analyses were performed using chi-square, McNemar’s, and Kaplan Meier tests.

**Results:**

After 12 months, the recall rate was 87%. Survival rates of CN and GP restorations were 92.5% and 97.7%, respectively. Three CN and one GP restorations lost retention. Seven CN (17.9%) and five (11.6%) GP restorations were scored as bravo for marginal adaptation and no significant difference was seen between groups (p=0.363). One (2.7%) CN and two GP (4.7%) restorations were scored as bravo for marginal discoloration, but no significant difference was observed between groups(p=1.00). For surface texture, three (8.1%) CN and three (7%) GP restorations were scored as bravo (p=1.00). None of the restorations demonstrated post-operative sensitivity or secondary caries at any examinations.

**Conclusion:**

The tested restorative materials performed similar successful clinical performances after 12 months. ClinicalTrials.gov (NTC04825379).

## Introduction

High plaque accumulation at the proximal surfaces of posterior teeth can lead to the development of caries and the necessity of dental treatments.^1 , 2^ The complexity of application methods for resin composites (RC) could influence the risk of secondary caries due to bacterial microleakage.^3^ Resin-based ion leaching materials such as resin-modified glass ionomer cements (RMGICs) have been used over the years to reduce recurrent caries^4^ since fluoride ions are released from these materials and mechanical properties are comparable with RCs.^5^ However, in modern dentistry, simplified, esthetic, and satisfactory restoration of tooth decay has led to innovative material developments. Rapidly cured restorative materials applied in large increments with self-adhesive properties became an important solution for easy and effective applications. Adhesion to tooth surface without additional procedures or conditioning has led to a single step placement that demands a short period of time. Furthermore, restorative materials with bioactive or caries-protective abilities have been introduced, such as highly viscous glass-ionomer cements (GICs).^6^ Recently, to overcome the limitations of restorative materials, it was attempted to add caries-protective ions, especially alkaline and alkaline earth ions, such as calcium, in addition to phosphate or fluoride.^7^ This newly introduced material contains alkalizing properties due to the release of hydroxyl ions. Caries lesions are caused by the imbalance of ions re-precipitated into tissues and ions that are released from the dental tissues. The calcium, fluoride, and phosphate releasing properties lead to apatite formations on tooth surface, which can explain their caries protective mechanisms.^8 , 9^ The monomer matrix of this new bioactive material, which consists of a mixture of urethane dimethacrylates, either aliphatic (UDMA) or aromatic-aliphatic, provides the alkasite characteristics. Moreover, studies have stated that this alkasite-based material has acid-neutralizing capabilities and prevents demineralization of enamel and dentin when subjected to lactic acid over a prolonged period.^8 , 9^

The alkasite-based tooth-colored material Cention N (Ivoclar Vivadent, Schaan, Liechtenstein) is considered integrant of a subgroup of RCs. This self-curing restorative, with optional additional light curing, displays a high polymer network density and degree of polymerization over the complete depth of the restoration.^10^ It was introduced as an amalgam replacing restorative material or as a white material that can compete to the physical properties of amalgam and bioactive properties of GICs.^11^

Several studies have demonstrated that this alkasite-based material releases acceptable levels of fluoride, calcium, and phosphate.^7 , 12 , 13^ A laboratory study reported that this material led to a reduction in enamel demineralization compared to a RC.^14^ Additionally, other studies on calcium releasing materials have shown volumetric expansion due to water sorption, potentially compensating for polymerization shrinkage.^15 , 16^ As a result, the use of this material may reduce the occurrence of gap formation between the tooth and the restoration.

Although several *in vitro* studies were performed on its mechanical properties since this material has been introduced, its clinical behavior has not yet been adequately monitored in clinical studies. Thus, the purpose of this randomized clinical trial was to assess the clinical performance of this alkasite-based restorative by comparing it with a RC in Class II cavities after 12 months. The null hypothesis was that there would be no significant difference between the clinical performance of the two restorative systems.

## Methodology

The Consolidated Standards of Reporting Trials (CONSORT) statement was followed to design the study.

### Ethics approval

The Ethics Committee of the institution approved the present clinical trial (KA-21046) and informed consent forms were taken from participants.

### Protocol registration

The study was registered at ClinicalTrials.gov (NTC04825379).

### Trial design and setting

This randomized, double-blind, controlled clinical trial was performed at the Department of Restorative Dentistry clinic.

### Sample size calculation

Power analysis using G* Power statistical software (ver. 3.0.10, Franz Faul, Universitat Kiel, Germany) was used to calculate the sample size. To achieve an w = 0.50 effect difference between the groups with 90% power and an alpha error of 5%, at least 26 restorations per group were needed. Considering the possibility of dropouts during follow-up, the sample size was increased to at least 50 in each group, and a total of 100 restorations were performed.

### Patient selection

**Figure 1:**
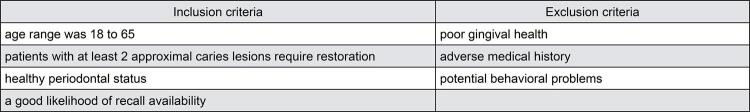
Inclusion and exclusion criteria for participants

Thirty-one patients with an average age of 33 years who fulfilled the inclusion and exclusion criteria were selected ( Figure 1 ). One of the researchers performed the assessments using a dental explorer, mouth mirror and periodontal probe.

### Randomization

Each patient received at least two restorations. Computer-generated tables were used to randomize restorative systems. A number was assigned to each restorative system in the tables for patient allocation. Only a researcher who was not involved in the study could access the tables.

### Restorative procedures

**Figure 2:**
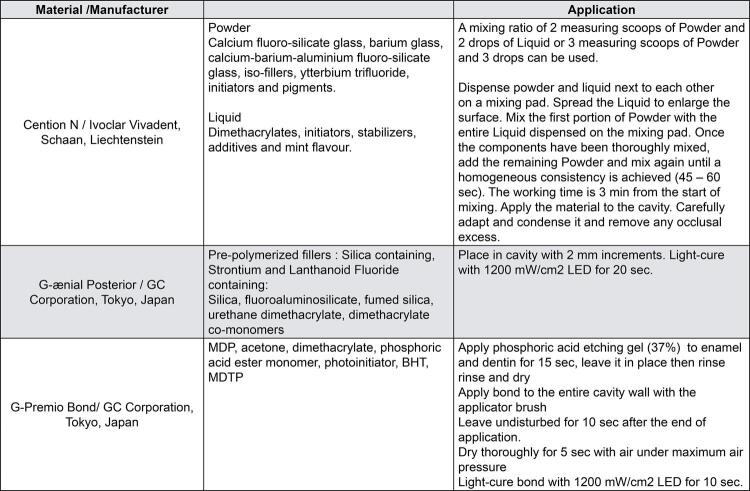
Applications of materials used in the study MDP: 10-Methacryloyloxydecyl dihydrogen phosphate, Bis-GMA: Bisphenol A diglycidylmethacrylate, HEMA: 2-Hydroxyethyl methacrylate, BHT: Butylated hydroxytoluene, MDTP: Methacryloyloxydecyl dihydrogen thiophosphate

One hundred restorations were placed in Class II cavities of 31 patients (17 males, 14 females) with an average age of 33 years. One week before the restorative procedures, dental prophylaxis was performed on participants and oral hygiene instructions were provided. All restorations were performed by the same researcher, who did not participate in the selection of study individuals. Teeth were cleaned with a slurry of pumice before preparations. Diamond fissure burs at high speed were used under water-cooling for preparations, whereas tungsten carbide burs with slow speed handpiece were used to remove carious tissues. If the patient felt pain or sensitivity, local anesthesia was applied. Tissue preserving cavity design was applied and the prepared cavities did not involve any cusps; additionally, the gingival walls were located supra gingivally. The cavities which did not meet these criteria were excluded. In deep cavities, a calcium hydroxide cavity liner (Life Regular Set, Kerr Corporation, Romulus, MI, USA) was placed. A sectional matrix was used before the application of restoratives. The teeth were restored either with an alkasite-based restorative (Cention N, Ivoclar Vivadent, Schaan, Liechtenstein [CN]) (n=50) or a RC (G-ænial Posterior GC, Tokyo, Japan [GP]) (n=50). Cotton rolls and saliva ejectors were used for isolation. The restorative materials used are shown in Table 2. Cention N was used without prior application of an adhesive system. Both restorative material systems were applied according to the manufacturers’ recommendations ( Figure 2 ).

Each increment of the RC (G-ænial Posterior) was light-cured for 40 seconds (at 1200 mW/cm^2^; Radii Plus; SDI, Bayswater, Australia). Finishing and polishing were done using flame-shaped fine finishing diamond burs and aluminum oxide discs (Optidisc, Kerr, Orange, CA, USA).

### Clinical assessments

Patients were called for controls after 1 week (baseline) and 6 and 12 months. The restorations were evaluated for the retention, marginal adaptation, marginal discoloration, surface texture, color match, and post-operative sensitivity according to the United States Public Health Service (USPHS) criteria.

Two researchers who were blinded to the group assignments and not involved in the clinical procedures evaluated the restorations. Ten representative photographs for each criterion were used to calibrate the researchers. Then, researchers assessed 10–15 restorations at two consecutive appointments. Intra- and inter-examiner agreement of at least 85% was necessary to begin the evaluation. Subjects were not informed about the group assignments either.

SPSS software (version 22.0; IBM Corp., Armonk, NY, USA) was used for statistical analyses. To compare the restorative materials, Pearson chi-square tests were conducted at each recall. Differences in the ratings of the two materials were assessed at 6 and 12 months. Cochran’s Q test was used to examine the changes over time for each material. McNemar’s test was used to compare the marginal adaptation, discoloration, and surface texture scores of each material with their baseline scores across various time points. Additionally, Kaplan-Meier analysis was performed to compare the survival rates of the restorations. The level of significance was set at p < 0.05.

## Results

**Figure 3:**
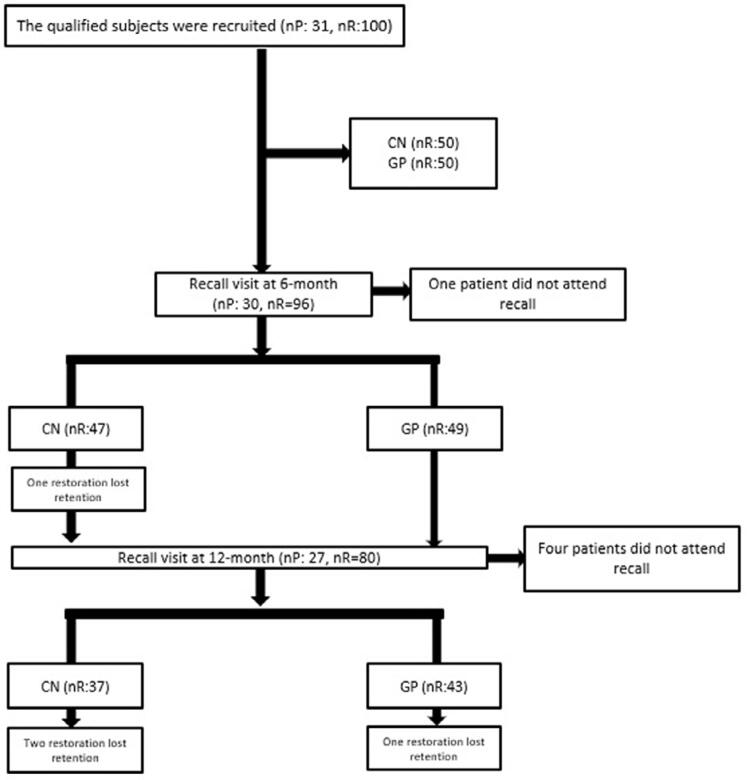
Flow diagram of the study

The flow chart was shown in Figure 3 . Recall rates for 6 and 12-month assessments were 96.7% and 87%, respectively. Clinical outcomes of tested groups were given in Table 1 .

**Table 1:** Clinical evaluation outcomes of different restorations

Evaluation	Score	Baseline n (%)		6-month n (%)		12-month n (%)	
Criteria		CN (50)	GP (50)	CN (47)	GP (49)	CN (37)	GP (43)
Retention	Alpha	50	50	47	49	37	43
		(100)	(100)	(97)	(100)	(94.9)	(97.7)
	Bravo						
	Charlie			1	0	2	1
				(3)	0	(5.1)	(2.3)
Marginal Adaptation	Alpha	50	50	39	48	30	38
		(100)	(100)	(83.0)	(98.0)	(81.1)	(88.4)
	Bravo			8s	1	7s	5s
				(17.0)	(2.0)	(18.9)	(11.6)
	Charlie						
Marginal Discoloration	Alpha	50	50	46	49	36	41
		(100)	(100)	(97.9)	(100)	(97.3)	(95.3)
	Bravo			1	0	1	2
				(2.1)	0	(2.7)	(4.7)
	Charlie						
Surface Texture	Alpha	50	50	44	48	34	40
		(100)	(100)	(93.6)	(98.0)	(91.9)	(93.0)
	Bravo			3	1	3	3
				(6.4)	(2.0)	(8.1)	(7.0)
	Charlie						
Color Match	Alpha	50	50	45	49	34	41
		(100)	(100)	(95.7)	(100)	(91.9)	(95.3)
	Bravo			2	0	3	2
				(4.3)	0	(8.1)	(2.7)
	Charlie						
Postoperative Sensitivity	Alpha	50	50	47	49	37	43
		(100)	(100)	(100)	(100)	(100)	(100)
	Bravo			0	0	0	0
				0	0	0	0
	Charlie						
Secondary caries	Alpha	50	50	47	49	37	43
		(100)	(100)	(100)	(100)	(100)	(100)
	Bravo			0	0	0	0
				0	0	0	0
	Charlie						

sIndicates significant difference in comparison with baseline according to Cochran’s Q test fallowed by McNemar's test (p<0.05) CN: Cention N, GP: G-ænial Posterior

The outcomes were scored as alpha: clinically very good, bravo: clinically good, acceptable, charlie: clinically unacceptable

One (3%) CN restoration lost retention after 6 months, and two (5.1%) CN and one (2.3%) GP restorations lost retention at 12-month evaluations.

Additionally, eight CN (17%) and 1 GP (2%) restorations exhibited bravo scores for marginal adaptation after 6 months and McNemar’s test exhibited changes in marginal adaptation in CN at 6 -month recall (p=0.016). CN showed significantly higher bravo scores than GP (p=0.015). After 12 months, 7 CN (17.9%) and 5 (11.6%) GP restorations were scored as bravo and no significant difference was seen between the groups (p=0.363). Four patients could not attend to the 12-month recall and 1 patient with a bravo score was among them. Both groups showed significant changes in marginal adaptation after 12 months (p=0.004, p=0.019).

One (2.1%) CN restoration showed bravo score, whereas all GP restorations were scored as alpha for marginal discoloration at 6-month examinations. At 12-month recall, 1 (2.7%) CN and 2 GP (4.7%) restorations were scored as bravo. However, no statistically significant difference was detected between groups after 12 months (p=1.00).

For surface texture, 3 (6.4%) CN and 1 (2.0%) GP restorations showed bravo scores at 6-month evaluations (p=0.357) whereas, 3 (8.1%) CN and 3 (7%) GP restorations were scored as bravo at 12-month recall (p=1.00).

In terms of color match, 2 (9.1%) CN restorations showed bravo scores at 6-month examinations. At 12-month recall, 3 (8.1%) CN and 1 (2.7%) GP restorations exhibited bravo scores (p=0.658), but no significant difference was seen between the groups at any evaluation point.

No post-operative sensitivity or secondary caries were seen at any recall assessment.

**Figure 4:**
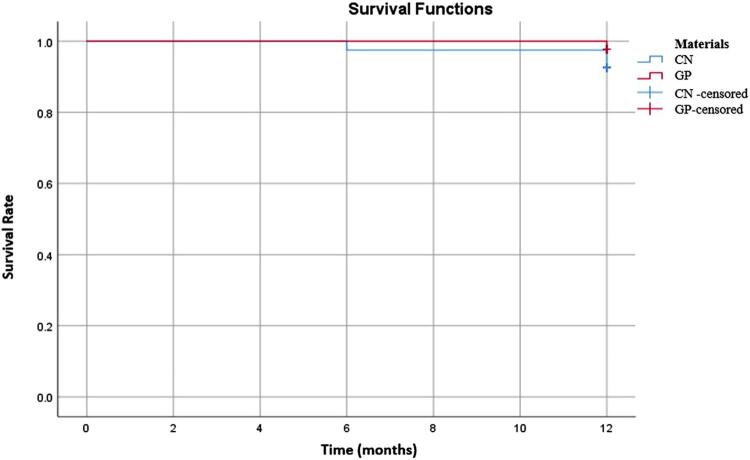
Survival curves for tested groups (CN [Cention N], GP [G- ænial Posterior])

The Kaplan–Meier analysis ( Figure 4 ) revealed no significant difference between the survival rates of the two tested restorative materials (Log rank: p=0.26). The 12-month survival rates of CN and GP were 92.5% and 97.7%, respectively.

## Discussion

New approaches in restorative materials were focused on the development of self-adhesive, dual-cured restorative materials that can be placed in bulk to simplify the placement of posterior restorations and have caries-protecting properties. The self-cure option leads to better depth of cure and these materials also have bioactive characteristics such as leaching remineralizing ions.^17^ CN is a bioactive, alkasite-based material with both dual- and self-cure properties which can be applied in one increment. Therefore, it can compete with bulk-fill RCs that can be placed easily with less increments in a short period of time. Previous laboratory studies stated that this material showed acceptable results for microleakage,^18^ shear bond strength,^19^ microhardness,^12 , 18 , 19^ and compressive strength.^18^ These characteristics could make it preferable as a long-term direct restorative material. In terms of flexural strength, the superiority of CN was shown when compared to a bulk-fill RC and Equia Forte HT Fill.^20^ Additionally, studies that compared the flexural strengths of CN and Zirconomer showed that CN had higher flexural strength values than Zirconomer.^20 , 21^ Mishra, et al.^22^ (2018) also stated that GIC and amalgam had lower flexural intensity than CN. This alkasite-based restorative material has a thick polymer network and the powder of this material consists of glass filler -barium aluminum silicate, ytterbium trifluoride, glass filler- calcium barium aluminum fluorosilicate, and glass filler- calcium fluorosilicate and alkaline.^23^ These fillers provide a sufficient strength to be a posterior restorative. In addition to these mechanical properties, the ion releasing (calcium, hydroxyl, and fluoride) properties can stabilize the oral pH and form apatite which reduces demineralization.^11^

Although several *in vitro* studies have been carried out on CN,^18 , 24 , 25^ this is, as far as we know, the first clinical study that compared this newly marketed alkasite-based restorative material in Class II restorations. Given the lack of information on the clinical behavior of this restorative material in Class II restorations, comparing this clinical trial with any other study done before is impossible. Also, until now, there has been no clinical study published that compared this alkasite-based restorative material with a RC that is commonly used for posterior teeth restoration. The present study assessed the clinical performance of the alkasite-based restorative material in comparison with a RC. The study hypothesis was partially rejected. Although both restorative materials showed similar performances, the alkasite-based material exhibited higher bravo scores for marginal adaptation than the posterior RC after six months. Despite the fact that the alkasite-based restorative material showed high bravo scores for marginal adaptation after six months, no increase were detected following this criterion after one year. Therefore, there was a stabilization after a year for marginal degradation. After one year, the alkasite-based restorative material showed comparable bravo scores to RC for marginal discoloration and surface texture. Therefore, the new material could be considered successful in terms of esthetic characteristics for posterior teeth after a year.

The only clinical trial published on the clinical performance of CN was in the restoration of non-carious cervical lesions by comparing it with a RMGIC.^26^ This clinical study was a split-mouth, randomized controlled trial and the authors evaluated the clinical performance of the restorations after 1 and 6 months and 1 year following the USPHS criteria,^26^ same as the present study. Both materials performed similarly after one month in retention and anatomic form. However, alkasite-based restorative CN showed significantly better results after 6 months and 1 year. The RMGIC restoration showed higher discoloration after 6 months and 1 year, whereas CN performed better regarding marginal integrity at all time intervals. The authors concluded that alkasite-based restorative material displayed superior technical, mechanical, and esthetic performance in a follow-up period of one year and can, therefore, be recommended as an alternative to RMGICs.^26^

In the present study, CN showed similar retention rate with the RC in 1-year follow-up. However, the marginal adaptation scores of CN significantly worsened after 6 months and RC showed statistically higher alpha scores than CN. On the other hand, the bravo scores of both groups were similar after one year.

After removing caries lesions in the proximal surfaces, the environment becomes less cariogenic, affecting the neighboring tooth as well. The ion-leaching restorative material can help to reduce bacterial growth and promote the formation of fluorohydroxyapatite in proximal caries lesions.^27^ Theerarath and Sriarj^28^ (2022) showed *in vitro* that CN increased the surface hardness of the adjacent tooth when compared to a RC. Additionally, the fluoride release level of CN was found similar to a RMGIC; Fuji II.^29^

Posterior RCs are desired by patients for esthetic reasons and preferred by the dentists for their advantages such as the superior protection of hard tissues with a conservative cavity preparation and cheaper price compared to porcelain restorations. Bulk-fill RCs have been used very often recently for their short application time and ensured deep polymerization.^30^ However, over time, marginal adaptation of RCs becomes poorer leading to microleakage and secondary caries.^30 , 31^ In addition, due to polymerization stresses, postoperative sensitivity is expected.^31^ The alkasite-based material can easily be applied in one increment, it leaches remineralizing ions, and presents higher physical properties.^11^

The introduction of alkasite-based restorative material was connected with the hope of replacing amalgam. Therefore, CN could be a more reliable restorative material in minimal intervention oral care based adhesive systems. To improve its mechanical properties, several attempts are still underway. Recently, a manageable self-mixing capsule has been developed for reassessment of the material properties; however, opportunities for improvement are still existing.

This study has some limitations that must be considered. Firstly, the short evaluation period. This study is a preliminary report and the evaluation period is not long enough. However, no clinical data on this material has been available to date. Therefore, studies examining the restoration of Class II cavities with longer follow-ups are needed. Secondly, this study included individuals with healthy periodontal tissues and, thus, studies with different participants should be performed in the future.

Lastly, in this study, cotton rolls were used for isolation to imitate clinical conditions. A meta-analysis study by Heintze, et al.^32^ (2015) showed that the rubber dam isolation is not viable for long term restorations. Daudt, Lopes and Vieira^33^ (2013) also reported that both rubber dam and cotton roll isolation led to similar clinical results. Finally, future *in vitro* and *in vivo* studies must be conducted to shed light on further clinical applications of this material.

## Conclusion

Clinical performances of the alkasite-based restorative and resin composite were similar and both materials showed a good survival after 12 months.
